# Disparities in Female Pediatric, Adolescent and Young Adult Oncofertility: A Needs Assessment

**DOI:** 10.3390/cancers13215419

**Published:** 2021-10-28

**Authors:** Leslie Coker Appiah, Yueyang Frances Fei, Mallery Olsen, Steven R. Lindheim, Diane M. Puccetti

**Affiliations:** 1 Division of Academic Specialists in Obstetrics and Gynecology, Department of Obstetrics and Gynecology, The University of Colorado School of Medicine, Denver, CO 80045, USA; 2 Pediatric and Adolescent Gynecology, Children’s Hospital Colorado, Denver, CO 80045, USA; 3 Pediatric and Adolescent Gynecology, Nationwide Children’s Hospital, Columbus, OH 43205, USA; fei.10@osu.edu; 4 Department of Medicine, The University of Wisconsin School of Medicine, Madison, WI 53705, USA; mallery.olsen@nationwidechildrens.org (M.O.); puccetti@pediatrics.wisc.edu (D.M.P.); 5 Pediatric Hematology/Oncology, American Family Children’s Hospital, Madison, WI 53705, USA; 6 Division of Reproductive Endocrinology and Infertility, Department of Obstetrics and Gynecology, Wright State University, Dayton, OH 45409, USA; steven.lindheim@wright.edu; 7 School of Medicine, Shanghai Jiao Tong University, Shanghai 200240, China

**Keywords:** adolescent and young adult, cancer, oncology, fertility, fertility preservation, disparities, access, oncofertility, reproductive health

## Abstract

**Simple Summary:**

The following review addresses the effects of cancer and cancer treatments on fertility and reproductive health, and reviews standard and novel fertility preservation options. This article presents a needs assessment focusing on disparities in access to care for the pediatric, adolescent, and young adult (AYA) population to include cost, provider bias, inequitable referral patterns to reproductive specialists, and a lack of knowledge within the medical community regarding assisted reproductive technologies and reproductive health care in survivorship. The information presented in this article is targeted to oncologists, gynecologists, pediatric subspecialists, and primary care providers who care for this population and introduces areas for further research to address gaps in care and improve access for this population.

**Abstract:**

Advancements in cancer screening and implementation of targeted treatments have significantly improved survival rates to 85% for pediatric and AYA survivors. Greater than 75% of survivors will live to experience the long-term adverse outcomes of cancer therapies, termed late effects (LE), that disrupt quality of life (QoL). Infertility and poor reproductive outcomes are significant disruptors of QoL in survivorship, affecting 12–88% of survivors who receive at-risk therapies. To mitigate risk, fertility preservation (FP) counseling is recommended as standard of care prior to gonadotoxic therapy. However, disparities in FP counseling, implementation of FP interventions, and screening for gynecologic late effects in survivorship persist. Barriers to care include a lack of provider and patient knowledge of the safety and breadth of current FP options, misconceptions about the duration of time required to implement FP therapies, cost, and health care team bias. Developing strategies to address barriers and implement established guidelines are necessary to ensure equity and improve quality of care across populations.

## 1. Introduction

Almost one million women are diagnosed with cancer each year in the United States. Ten percent are women under the age of 40, including more than 48,000 new cancer diagnoses in adolescent and young adult (AYA) women ages 15 to 39 years and approximately 5000 new diagnoses in pediatric girls between the ages of birth through 14 years [1,2]. Cancer treatments can lead to primary ovarian insufficiency (POI), infertility, and long-term cardiovascular, cognitive, and skeletal risks associated with menopause. As oncologic care advances and survival improves, fertility preservation (FP) becomes a vital consideration at diagnosis and in survivorship. Fertility potential depends on age, baseline fertility, and treatment regimen [3,4]. Despite the recommendations that providers address the possibility of infertility with proposed cancer treatments and refer patients to specialists for further management, disparities persist in oncofertility care at diagnosis and in survivorship [5,6]. This review will discuss existing barriers and considerations to improve provider knowledge, reduce bias and increase access to equitable care. 

### Cancer Treatment and Impact on Female Fertility 

Currently, there are estimated to be more than 500,000 survivors of childhood cancer living in the United States, 75% of whom will experience late effects (LE) of therapy [1,2,7]. POI is a reproductive LE affecting 12% of female childhood cancer survivors treated with alkylating agents and radiation [8]. A greater proportion will experience diminished ovarian reserve (DOR) leading to infertility. Additionally, young adult survivors treated between the ages of 18 and 39 years will be 40% less likely to conceive than age matched controls [9].

Alkylating agents such as cyclophosphamide cause vascular toxicity to reproductive organs as well as direct DNA damage to growing and dormant cells. This leads to menstrual changes, acute ovarian failure, and DOR [5]. Other agents such as platinums, taxanes, anthracyclines, and topoisomerase inhibitors cause ovarian damage to a lesser extent but are still important to consider as potential causes of decreased fertility. The cyclophosphamide equivalent dosage (CED) calculator uses a patient’s dosage, body weight, and specific treatment regimen to calculate a normalized dosage to allow better comparison of commonly used alkylating regimens and their impact on fertility. For example, a CED greater than or equal to 8000 mg/m^2^ has a high level of increased (greater than 80%) risk of premature ovarian insufficiency and infertility in the post-pubertal patient [10,11,12,13,14].

Radiation therapy (RT) also increases the risk of infertility, depending on a patient’s age, pre-treatment ovarian reserve, total radiation dose, and fractionation schedule. Cranial radiation disrupts hypothalamic and pituitary function resulting in oligomenorrhea and hypogonadism. Direct ovarian radiation exposure causes menstrual irregularity, DOR, and POI. Scatter doses of radiation from abdominopelvic or craniospinal RT can cause ovarian failure in 50–70% of cases [12]. Direct pelvic radiation to the uterus causes myometrial fibrosis, diminishes uterine volume, and thins the endometrium leading to abnormal placentation, miscarriage, preterm birth, and abnormal fetal growth [3,15]. Studies show that direct radiation doses >25 Gy to the uterus in childhood appears to induce irreversible damage [15,16,17].

Historically, risk stratification systems have used the categories of low (<20%), moderate (≥30–70%), and high (>80%) to stratify the risk of ovarian failure after therapy [18]. Table 1 lists common chemotherapeutic agents and associated gonadotoxic risk based on historical risk stratification systems [13,19,20]. In 2020, the Pediatric Initiative Network of the Oncofertility Consortium developed and published a new stratification system to better describe risk, using the terms minimally increased risk, significantly increased risk and high level of increased risk to replace the previous categories (Table 2) [14]. The reasons for the new classification system were to demonstrate that low-risk therapies also result in some harm and to stratify by pubertal status since age confers protection due to a greater number of baseline follicles in younger patients.

## 2. Fertility Preservation Methods

Over the past decade, there have been significant advancements in FP stem cell research and cryopreservation technology. A wide variety of methods are now available for the newly diagnosed patient. No standard method is applicable to every patient, so an individualized approach is needed. 

### 2.1. Established Technologies

Embryo and mature oocyte cryopreservation are well-established technologies with success rates of 40–70% in adult women less than 35 years who do not have cancer (https://www.sartcorsonline.com/Predictor/Patient, accessed on 6 June 2021). However, less is known about success rates in adolescents and cancer survivors. Assisted reproductive technologies (ART) require a post-pubertal patient who can undergo ovarian stimulation with gonadotropins. After stimulation, mature oocytes are then harvested and cryopreserved with or without fertilization until future use. Ovarian stimulation and retrieval require an average of 12 days and may not be feasible for patients who require urgent treatment initiation such as those with leukemia or mediastinal masses from lymphoma affecting cardiovascular and pulmonary function [3,21,22].

Patients without a partner are counseled that oocyte cryopreservation maintains reproductive autonomy until the patient identifies a partner or elects to use donor sperm to create embryos. Ovarian stimulation is increasingly being used in adolescents ages 13–21 years. A recent study shows no statistically significant difference in the total gonadotropin dose, number of days stimulated, and number of mature oocytes retrieved and frozen in immediately post-pubertal patients compared to older adolescents and young adults [23]. Children who elected ovarian stimulation have not reached the age to utilize the frozen oocytes, and therefore outcomes including malformation risk, fetal loss and live birth rates remain unknown.

Ovarian transposition and ovarian shielding are well-established techniques with efficacy rates up to 90% in women <40 years [24,25]. However, several studies report underutilization of these techniques, which can often be performed in conjunction with other FP methods, such as ovarian tissue cryopreservation (OTC) [12]. These methods can protect against radiation but are ineffective against chemotherapy. Success rates depend on the patient’s age, radiation dose, location of transposition and whether concurrent chemotherapy is administered.

OTC is the only non-experimental option available to pre-pubertal girls. Due to the widespread utilization of this method internationally and consistent with European Society of Human Reproductive and Embryology (ESHRE), European Society for Medical Oncology (ESMO) and ASRM guidelines, the experimental label has been removed and OTC is now standard of care [4,26,27]. No hormonal stimulation is needed and OTC may be performed within days of the initial FP consult [28]. This method has seen exponential growth over the past 15 years with over 200 live births after autografting and live birth rates of 29–41% [3,29]. The majority of cases have occurred in women from whom OTC was harvested post-puberty. Between 2007 and 2017, the median age of patients utilizing OTC dropped from 30.6 years to 12.4 years of age. The youngest patient to undergo ovarian harvest with a subsequent live birth in adulthood was a prepubertal 9-year-old who underwent bone marrow transplant for beta-thalassemia [30,31]. Ovarian tissue can be grafted to heterotopic locations, including the upper abdomen or extra-abdominal location. However, heterotopic transplantation requires ART for conception and live birth rates have been low [32,33]. Autografting to any site potentially preserves ovarian function throughout the lifetime of the graft, with added benefits on cardiovascular and bone health [28]. The average duration of hormone production from grafted ovarian tissue is estimated to be 5 years per graft [29]. Table 3 summarizes standard and investigational FP options with associated costs.

### 2.2. Investigational Techniques

Investigational techniques include in vitro maturation (IVM) of the immature oocyte and ovarian suppression. IVM is available to pre- and post-pubertal girls and women since no hormonal stimulation is required. Therefore, there is also no delay in initiating chemotherapy [5,11]. Ovarian suppression is only feasible in post-pubertal patients. 

Retrieval of immature oocytes for IVM obviates the need for a partner and can be performed at time of ovarian tissue harvest for OTC. In IVM, immature oocytes and primordial follicles are harvested at early stages of development and matured in vitro for cryopreservation and future fertilization. A few human live births have been reported from IVM of growing oocytes following cancer treatment [34,35]. However, there have been no live human births reported with IVM from immature primordial follicles despite success in the mouse model [36].

Ovarian suppression using gonadotropin-releasing hormone agonist (GnRH-a) therapy is hypothesized to result in ovarian quiescence with decreased blood flow to the ovaries and decreased sensitivity to chemotherapy. GnRH-a therapy for ovarian protection is still considered experimental but may be a reasonable option for post-pubertal patients when other treatments are not feasible, such as when emergent chemotherapy is necessary. Ferto-protective effects of GnRH-a therapy are most well-studied in the breast cancer population; a meta-analysis comparing five randomized studies showed a dramatic difference between early menopause (31% in control vs. 14% in ovarian suppression groups) and pregnancy rates (5.5% vs. 10.3%). There was no difference in overall or disease-free survival between the groups [37]. However, studies in other populations have shown conflicting results, with some systematic reviews and meta-analyses showing ovarian protection while others show no increase in pregnancy rates after GnRH-a therapy [38,39]. In a meta-analysis of 12 studies, Chen et al. concluded that “GnRH agonist appears to be effective in protecting the ovaries during chemotherapy, in terms of maintenance and resumption of menstruation, treatment-related premature ovarian failure and ovulation. Evidence for protection of fertility was insufficient and needs further investigation” [40]. ASCO and the National Comprehensive Cancer Network (NCCN) recommend that GnRHa therapy be used for ovarian protection during chemotherapy in the absence of available standard FP options [41,42]. 

One of the concerns with GnRH-a therapy is the potential flare effect resulting in a rise in gonadotropins and sex steroids that occurs when administered in the follicular phase of the menstrual cycle [43]. This may cause bleeding which can be problematic in patients with pancytopenia. Additionally, there is a theoretical concern that this increase in gonadotropins may also enhance blood flow to the ovary, increasing ovarian exposure to chemotherapeutic agents. The average time to achieve complete suppression of the hypothalamic-pituitary-ovarian (HPO) axis is 2 weeks and oral progestin therapy concurrently with GnRH-a therapy has been shown to minimize this effect. When given as part of ovarian stimulation protocols, the GnRH-a flare effect is mitigated with aromatase inhibitors [44].

### 2.3. Fertility in Survivorship

Ovarian reserve testing in survivorship is an important aspect of fertility care as it allows an opportunity to pursue fertility in survivorship. Post-treatment FP is particularly salient for patients who required emergent therapy and could not pursue FP or declined FP due to concerns about hormone exposure or potential delays. Children’s Oncology Group (COG) Long Term Follow-up (LTFU) Guidelines recommend assessing follicle stimulating hormone (FSH) and estradiol for post-pubertal children with menstrual cycle dysfunction suggestive of POI and anti-Mullerian hormone (AMH) to identify DOR. At-risk patients who desire fertility should be referred to reproductive endocrinology and infertility (REI) specialists for ovarian reserve testing and options counseling. 

Antral follicle count (AFC) is an established direct measure of ovarian reserve and is obtained by adding the number of follicles measuring 2–10 mm in both ovaries by ultrasound. An AFC < 6 is diagnostic of DOR, and predicts low response to ART and low live birth rates [45,46]. AFC is more precise when performed by transvaginal ultrasound, which can be challenging in adolescents or survivors with post-treatment vaginal narrowing thereby limiting utility. Further, AFC is not readily available due to a lack of adequately trained sonographers. Training sonographers to perform this measurement can improve access to fertility assessment by a general gynecologist, minimizing the cost of specialty care. 

AMH is a glycoprotein expressed at 36 weeks’ gestation in the pre-antral and small antral follicles of the ovary and thus reflects function of the primary and secondary follicles [47,48]. AMH controls folliculogenesis by inhibiting initial recruitment of follicles and is independent of the menstrual cycle. Levels are near undetectable at birth with a postnatal rise at 3 months of age [49]. Values fall thereafter and exhibit a subtle rise within the first 2 to 4 years of life. Maximum levels are achieved in early puberty and plateau until age 25 when levels begin to decline. Normal values range from 1 to 3.5 ng/mL and values of 0.5–1.0 ng/mL are consistent with DOR and suggest a shortened reproductive window [50]. A value of <0.5 ng/mL is considered very low and suggests impending POI and low response to ovarian stimulation. In patients with a high risk of ovarian damage from planned therapy, AMH can be used to assess baseline ovarian reserve prior to treatment for counseling purposes [51,52]. Limitations of AMH include intrapersonal fluctuations that can be observed over the course of years and a historical lack of comparison between assays. Standardization of current assays has resolved the latter concern. AMH provides an index of treatment gonadotoxicity, and allows comparison of different treatment regimens, but the data cannot be extrapolated to fertility, and data regarding the use of AMH to estimate time to menopause after cancer is limited [53]. Therefore, although AMH correlates with AFC and reliably predicts response to ART, AMH does not reliably predict live birth rates [48,54]. 

## 3. Barriers to Care

Despite the increasing awareness of the effects of cancer therapies on fertility, and an increase in FP referrals for pre-adolescent and adolescent patients, overall referral and utilization of these services remains low [55,56,57,58]. Referral rates range from 1.7% to 3% for reproductive-aged patients with any cancer diagnosis in the United States compared to European data which shows only slightly higher rates of referral (9%), falling short of international guidelines [58,59,60]. Concerningly, referrals are inversely correlated with patient demographics, prognosis, advancing reproductive age, prior parity, and advanced disease. Patients’ lack of awareness of FP services, time pressures and conflicting priorities of physicians, low reimbursement, and a lack of collaborative multi-disciplinary approaches continue to hinder timely referrals [61,62]. These barriers are compounded in marginalized populations such as LGTQB patients with cancer. These patients report discrimination from health care providers by denying them access to FP information and services, mis-gendering, treating them disrespectfully, and assuming they are not interested in biological parenthood due to bias from providers [63,64,65].

### 3.1. Ethical Considerations

Patient autonomy is central to shared decision making. However, significant disparities in oncofertility care stem from paternalism and provider bias and lead to loss of patient autonomy. Multiple studies have shown survivors desire to have biologic children after therapy with one study reporting that 76% were interested in having children in the future [66]. Surveys have shown that infertility can become a significant cause of distress, depression, and decreased quality of life, which may persist long after cancer treatment and remission. Many patients confirm that this may be even more distressing than the cancer diagnosis or treatment itself. Patients also report less regret after counseling, even if FP options are ultimately not pursued, illustrating the importance of autonomy in patient decision making [67,68]. Despite the data, less than 50% of patients recall having a discussion about FP with their provider and less than 30% of patients who are referred pursue FP options [69,70]. Barriers to care include a lack of provider and patient knowledge of the safety and breadth of current FP options, the perception that FP delays cancer therapies, cost and health care team bias. Bias presents as concern that FP counseling is futile for patients with limited financial resources or poor prognosis and thus should not be offered. These barriers prevent FP counseling from being offered equitably across all populations. 

As the options for FP develop, it is also important to consider and discuss specific ethical issues. Parents of children may express concern that undergoing FP may provide false hope since ART does not always result in live birth [71]. Established methods such as oocyte and embryo freezing are not “insurance” or a “guarantee” that future biological children will be possible. Using gametes for future fertility following experimental methods also present ethical challenges. This is especially relevant for the pre-pubertal population where OTC and IVM are the only options available. Counseling regarding FP is often performed in the setting of a new stressful cancer diagnosis with treatment planned to start immediately. This raises the question of whether time can be spared to investigate or pursue any FP options. This is also a difficult time to discuss disposal of tissue in the event the patient does not survive. Many other legal issues need to be addressed, such as ownership of embryos, oocytes, and cryopreserved tissue, use of tissue for research, and possibility of donation of embryos/oocytes [72]. 

Religious beliefs also impact FP decision making and should be considered (Table 4) [73]. Judaism, Christianity, Islam, Hinduism, and Buddhism hold bioethical concepts regarding fertility and at the patient’s request, religious leaders and family members may actively participate in discussions concerning the use of ART. Religious views may often conflict with FP options for procreation, raising religious questions that may not have clear answers. Familiarity with religious practices surrounding FP is thus important when approaching the patient.

Assent is an important consideration in the pediatric population. As this population is unable to give consent due to age, assent should be assured prior to undergoing FP [74]. Parental and patient wishes may also be at odds with each other which can elicit conflict at the time of cancer diagnosis and many years later when the patient is cancer-free. Survivors may also feel undue or perceived pressure to use the tissue in later years. Optimal FP counseling requires awareness and sensitivity to bio-ethical standards, religious beliefs, legal issues surrounding the consent/assent process and end-of-life reproduction. FP therapies cannot be equitably offered to patients without appropriate and diligent attention to such concerns. 

### 3.2. Cost 

Insurance coverage of infertility and FP services varies significantly across the United States. Typically, when insurers cover IVF services, elective oocyte cryopreservation and medically indicated FP services are not included. Costs for oocyte, embryo, and OTC can range from $8000 to $20,000 with yearly storage costs of $275–$500 depending on whether the tissue is stored within an infertility practice or at a long-term storage site [75]. Ovarian suppression with GnRH-a can also cost up to $500 per monthly injection. Advocacy groups have worked to improve state mandated insurance coverage, but cost continues to be a significant barrier. In 2016, coverage for infertility was still in its infancy and no US states had laws to mandate insurance coverage for FP services to patients who were not yet infertile. As of 2021, 11 states (CA, CO, CT, DE, IL, MD, NH, NJ, NY, RI, UT) have enacted coverage for FP for patients who are at risk of infertility due to medically indicated treatment, with ongoing legislation in several other states [76]. However, these state-mandated laws are variable in coverage of procedures, fees, and number of cycles. Religious organizations, government insurance, and employers with <25–50 employees are often exempt from these laws. 

As of now, IVM and ovarian suppression with GnRH-a therapy solely for FP are not covered by most insurances as they are still considered experimental. However, GnRH-a therapy is often concurrently administered for menstrual suppression and is covered by insurance in this instance. OTC and ovarian transposition are often performed concurrently with other procedures under anesthesia to help defray cost. However, out-of-pocket expenses can reach upwards of $25,000 per procedure and thus are prohibitive to most patients. OTC is increasingly being covered by both private and government insurances, improving equity in care. 

### 3.3. Provider Knowledge 

#### 3.3.1. Fertility Preservation Options 

To expand use of FP services prior to cancer treatment, patients and providers must be aware of all available treatment options. A 2011 study of pediatric oncology providers reported that only 44% of respondents were familiar with ASCO recommendations regarding FP [77]. Greater than 90% of all respondents agreed that pre- and post-pubertal male and female patients should be counseled about potential impacts of treatment on future fertility. However, only 46% of respondents reported referring pubertal males to FP specialists more than half the time and only 12% reported referring pubertal females more than half the time. 

Similarly, a 2012 qualitative study of patients and oncologic health care providers also showed gender differences regarding information provided and referrals to reproductive specialists. Males are more likely to be provided information regarding potential impact on fertility and option for sperm banking. Health care providers reported less comfort in discussing FP options with females and withholding information due to concerns for urgent cancer treatment without delay [67]. One consequence of this knowledge gap and delay in referral is that few patients pursue FP services despite desiring future fertility options. In fact, thirty percent of male patient’s bank sperm when facing potentially gonadotoxic therapy, whereas only 10–25% of female patients elect FP [78]. 

#### 3.3.2. Safety and Timing of FP Interventions

Balancing the timeline of FP prior to cancer treatment is a major hurdle that requires knowledge of options, availability of specialists, and communication between providers. Conventional start controlled ovarian stimulation (COS) protocols required waiting until the start of the follicular phase which could delay treatment for 4–6 weeks. For this reason, oncologists have historically not referred all eligible patients for FP services. Random-start COS protocols are now standard of care for patients with cancer. This approach limits delays in initiation of therapy as timing of the patient’s menstrual cycle is irrelevant. Random-start COS may be performed in the early or late follicular or luteal phase. Late-follicular-phase protocols begin after menstrual cycle day 7 with emergence of a dominant follicle (>13 mm) [79]. In luteal-phase protocols, COS is initiated in the first 2–3 days of the early luteal phase. Studies show that while random-start COS results in longer length of ovarian stimulation by ~2 days (*p* < 0.001) and requires higher average gonadotropin use (4158 vs. 3404 IU/day, *p* = 0.002), there may be an advantage to random-start COS with respect to time to treatment for cancer [79]. Conversely, Moravek et al. showed that random-start COS does not hasten time to chemotherapy start and the average time of stimulation remained at approximately 2 weeks [78]. Further, when comparing follicular- to luteal-phase stimulation, random-start protocols have not impacted the number of oocytes retrieved or fertilization rates [80]. This is consistent with the emerging concept that follicular recruitment occurs in multiple waves during each menstrual cycle [81]. With timely referral, ovarian stimulation and retrieval can occur within 12 days of cancer diagnosis. Ovarian transposition, ovarian shielding, ovarian suppression, and OTC can be implemented without any delay but require a team of specialists knowledgeable in these technologies. Due to a lack of infrastructure and experienced providers, many institutions are unable to provide more recent technologies such as OTC and IVM; therefore, referrals to nearby institutions are necessary, which may delay time to treatment or increase costs of treatment [30]. Establishing regional centers of excellence with standard operating procedures may be an opportunity to address this barrier.

Provider knowledge regarding safety of FP in high-risk populations also presents challenges in providing equitable care. Patients with estrogen-sensitive cancers may not be referred for FP services due to the concern that increased estrogen levels may worsen prognosis. In conventional COS protocols, estrogen levels are high, which may promote tumor growth and increase progression of disease. However, newer protocols involving aromatase inhibitors or estrogen receptor inhibitors have been developed to address this concern [82]. Tamoxifen, an estrogen agonist-antagonist with anti-estrogen effects at the breast and central nervous system, is commonly used for treatment and chemoprophylaxis of hormone-sensitive breast cancers. Aromatase inhibitors such as letrozole suppress the production of estrogen by inhibiting the peripheral conversion of androgens to estradiol by aromatase. Tamoxifen and letrozole have been used with gonadotropins in ovarian stimulation cycles to prevent increased serum estradiol levels. Protocols with letrozole have shown higher oocyte and embryo yields compared with tamoxifen, so letrozole is used most [83]. Studies with letrozole have reported a similar number of retrieved and fertilized oocytes compared to standard ovarian stimulation protocols and estradiol levels are similar to natural cycles [84]. Aromatase inhibitors have thus been used safely in young adult patients with breast and endometrial cancer [85,86]. Recent studies on long-term outcomes show no difference in recurrence and survival outcomes even when aromatase inhibitors are not used [78,87]. 

### 3.4. Ovarian Reserve Testing and Pregnancy

Despite the growing body of knowledge around ORT, there remains no single best approach for monitoring ovarian reserve after gonadotoxic therapy and thus many patients lose the opportunity to pursue fertility in survivorship [88,89,90]. Based on the best available evidence, a reasonable approach to ovarian reserve monitoring may be to perform AMH and FSH 1-year post-treatment and refer to REI based on the values [50,89,91,92]. If FSH levels are <10 mIU/mL and/or AMH is ≥1 ng/mL, and thus normal, providers may consider retesting every 6–12 months if the patient is not interested in pursuing ART at that time. If FSH is ≥10 mIU/ml and/or AMH is <1 ng/ml suggestive of impending POI and diminishing reserve, referral to REI is indicated. The benefit of this approach is that it may allow screening by the primary multi-disciplinary team of oncologists, endocrinologist, or primary care provider with early and timely referral to REI [88,91]. It is important that patients be referred to a fertility specialist at any time they desire fertility or FP regardless of AMH and FSH levels. 

Conception in survivorship is typically safe and is dependent on several factors, including maternal health, recurrence risk and recovery of ovarian function. The average time to monitor for relapse is 2 to 5 years and recurrence rates vary depending on tumor characteristics, stage of disease and treatments received. Although a safe interval between completing chemotherapy and conceiving has not been established, it is known that the follicular life cycle is approximately 6–12 months, a time during which exposure to gonadotoxic therapies could provoke maximal mutagenesis [93]. Pregnancy outcomes in cancer survivors have also shown increased rates of preterm labor and small for gestational age infant when conception occurs within 12 months of therapy [94]. Therefore, expert consensus recommends waiting 12 months after completion of therapy to pursue fertility. It is well established that the offspring of cancer survivors do not have an increased incidence of fetal chromosomal or congenital abnormalities after chemotherapy compared to the general population and patients should be counseled as such [95,96]. Despite what is known, pregnancy may be discouraged by health care providers due to a lack of knowledge about safety and outcomes. Similarly, the risks and benefits of contraception during treatment and in survivorship also may not be well understood by oncologists and primary care providers necessitating discussion with a gynecologist. Patients who decline contraception should be advised to abstain during cancer therapy or use barrier contraception due to the teratogenic effects of cancer therapies in the first trimester. Patients should be counseled that the typical use failure rate of barrier methods is 12–21% [97]. Additionally, chemotherapy can be excreted in saliva and semen for 48–72 hours after a treatment, and therefore patients should either abstain during this time or use barrier methods [98,99]. Evaluation by a gynecologist or reproductive specialist at the end of treatment is important to address and dispel misconceptions regarding contraception and pregnancy safety. 

Multi-disciplinary teams can address many of the barriers to providing equitable oncofertility care by implementing models of care [100]. Given the ever-increasing health care demands, imbalanced medical resource distribution, inadequate health insurance, and unsatisfactory implementation of established guidelines, individual professional or discipline knowledge is no longer sufficient to address the full scope of needs for this patient population. Thus, the use of multi-disciplinary collaborative guidance documents such as diagnosis specific FP management guidelines, consultation systems, and protocols related to multi-disciplinary comprehensive management for difficult and severe diseases is warranted [101]. The goal is timely and effective diagnosis and treatment. Models such as the EU-REFER model of care may serve as a template to enhance the awareness, create stream-line processes, and increase the utilization of fertility sparing options among health care professionals and AYA cancer patients. 

## 4. Conclusions 

FP counseling is standard of care for all patients of reproductive age who are newly diagnosed with cancer and continued efforts to dismantle barriers to care are necessary in ensuring equity. Random start protocols and designation of OTC as clinical care in pre- and post-pubertal women are advances in the field that need to be broadly implemented within cancer centers. Validated protocols for monitoring ovarian reserve in survivorship remain necessary to identify survivors at risk of POI and improve post-treatment fertility rates. Regional centers of excellence may facilitate implementation of guidelines and improve access for underserved populations. Finally, continued collaboration between advocacy groups, academic medical centers and state and federal legislatures will improve access to FP services for all patients.

## Figures and Tables

**Table 1:** Chemotherapeutic Agents and Risk of Gonadotoxicity.

High Risk	Medium Risk	Low or No Risk	Unknown Risk
Nitrogen mustard Chlorambucil Cyclophosphamide Melphalan Busulfan Procarbazine Dacarbazine Doxorubicin Carmustine Lomustine	Vinblastine Cytosine arabinoside Cisplatin Carboplatin	Methotrexate 5-Fluorouracil 6-Mercaptopurine Vincristine Bleomycin Actinomycin D	Paclitaxel Taxotere Oxaliplatin Irinotecan Trastuzumab Pertuzumab Certuximab Erlotinib Daunorubicin Imatinib

**Table 2:** New Female Risk Stratification System.

Female Risk Stratification			Minimally Increased Risk	Significantly Increased Risk	High Level of Increased Risk
Alkylators CED gm/m^2^		Prepubertal	CED < 8 gm/m^2^	CED < 8 gm/m^2^	CED > 12 gm/m^2^
		Pubertal	CED < 4 gm/m^2^	CED 4–8 gm/m^2^	CED > 8 gm/m^2^
Heavy Metal mg/m^2^			Cisplatin Carboplatin		
Hematopoietic Stem Cell Transplant					Alkylator +/− Total body irradiation Myeloablative and Reduced intensity regimens
Radiation	Ovary	Prepubertal		<15 Gy	≥15 Gy
		Pubertal		<10 Gy	≥10 Gy
	Hypothalamus		22–29.9 Gy	30–39.9 Gy	≥40 Gy

CED = Cyclophosphamide equivalent dose; Gy = Gray; Meacham L et al. Adol Young Adult Oncol May 2020 [14]; Pediatric Initiative Network of the Oncofertility Consortium.

**Table 3:** Fertility Preservation Options and Costs.

Fertility Preservation Options and Costs					
Standard	Success Rates	Costs	Age	Investigational	Age
Mature oocyte cryopreservation	35–50% under age 35	$8–10k	Post-pubertal and older	Immature oocyte cryopreservation	All
Sperm cryopreservation (ejaculate, TESA, TESE)		$400–$500	Post-pubertal and older	Testicular tissue freezing	Pre-pubertal
Embryo cryopreservation	60–70% under age 35	$12–20k	18 years and older	GnRHa ovarian suppression	Post-pubertal
Ovarian transposition	60–90%	Covered by insurance	All	Annual storage costs for gametes and tissue average $275–$500	
Ovarian shielding	75–80%	Covered by insurance	All		
Ovarian tissue freezing	29–41%	Covered by insurance	All		

**Table 4:** Religious views on fertility preservation therapies.

Religion	AID	AIH	IVF	Oocyte/Embryo Donation	Gestational Carrier
**Buddhism**	**A**	**A**	**A**	**A**	**A**
**Church of England**	**A**	**A**	**A**	**A**	**A**
**Hinduism**	**A**	**A**	**A**	**A**	**A**
**Islam**	**D**	**A**	**A**	**D**	**D**
Judaism *	**D**	**A**	**A**	D **	D **
**Roman Catholicism**	**D**	**A ^+^**	**D**	**D**	**D**

Approve = A; disapprove = D; AID = artificial insemination by donor; AIH = artificial insemination by husband; * AID is permitted in Judaism if the sperm is not wasted; ** Some rabbis permit leniency in practice and may allow if the oocyte is from a Jewish woman or the surrogate is of Jewish ancestry; ^+^ Acceptable if the sperm is collected via intercourse.
